# Deletion of Mettl3 at the Pro-B Stage Marginally Affects B Cell Development and Profibrogenic Activity of B Cells in Liver Fibrosis

**DOI:** 10.1155/2022/8118577

**Published:** 2022-06-14

**Authors:** Xinmei Kang, Shuhan Chen, Lijie Pan, Xiaoqi Liang, Di Lu, Huaxin Chen, Yanli Li, Chang Liu, Mian Ge, Qi Zhang, Qiuli Liu, Yan Xu

**Affiliations:** ^1^Biotherapy Center, The Third Affiliated Hospital, Sun Yat-sen University, Guangzhou, China; ^2^Cell-Gene Therapy Translational Medicine Research Center, The Third Affiliated Hospital, Sun Yat-sen University, Guangzhou, China; ^3^Department of Anesthesiology, The Third Affiliated Hospital, Sun Yat-sen University, Guangzhou, China; ^4^Guangdong Provincial Key Laboratory of Liver Disease Research, The Third Affiliated Hospital, Sun Yat-sen University, Guangzhou, China

## Abstract

N6-methyladenosine (m^6^A) modification plays a pivotal role in cell fate determination. Previous studies show that eliminating m^6^A using *Mb1-Cre* dramatically impairs B cell development. However, whether disturbing m^6^A modification at later stages affects B cell development and function remains elusive. Here, we deleted m^6^A methyltransferase Mettl3 from the pro-B stage on using *Cd19-Cre* (*Mettl3* cKO) and found that the frequency of total B cells in peripheral blood, peritoneal cavity, and liver is comparable between *Mettl3* cKO mice and wild-type (WT) littermates, while the percentage of whole splenic B cells slightly increases in *Mettl3* cKO individuals. The proportion of pre-pro-B, pro-B, pre-B, immature, and mature B cells in the bone marrow were minimally affected. Loss of *Mettl3* resulted in increased apoptosis but barely affected B cells' proliferation and IgG production upon LPS, CD40L, anti-IgM, or TNF-*α* stimulation. Different stimuli had different effects on B cell activation. In addition, B cell-specific Mettl3 knockout had no influence on the pro-fibrogenic activity of B cells in liver fibrosis, evidenced by comparable fibrosis in carbon tetrachloride- (CCl_4_-) treated *Mettl3* cKO mice and WT controls. In summary, our study demonstrated that deletion of Mettl3 from the pro-B stage on has minimal effects on B cell development and function, as well as profibrogenic activity of B cells in liver fibrosis, revealing a stage-specific dependence on Mettl3-mediated m^6^A of B cell development.

## 1. Introduction

The development and maturation of B cells are tightly regulated processes that involve several steps [1–3]. First, bone marrow resident common lymphoid progenitor cells (CLPs) commit to the B cell lineage and enter the pro-B cell stage under the control of crucial transcription factors E2A, EBF, and Pax5 [4]. Then, heavy-chain DJ and VDJ rearrangements of immunoglobulin-gene happen in pro-B cells. When they start to express light-chain, the pro-B cells progress to the pre-B stage [5–7]. Once finishing VJ rearrangement of the light chain and expressing IgM, the cells become immature B cells. Then, the immature B cells undergo further negative selection, and the survival cells upregulate the B cell-activating factor receptor (BAFF-R) and acquire survival signals from BAFF. The survival signals support their survival when they exit bone marrow, enter the circulation, and migrate to the spleen for further maturation [4, 8, 9]. Growing evidence indicates that B cell differentiation is controlled by complex epigenetic and transcriptional programs [10–13].

N6-methyladenosine (m^6^A) modification is the most abundant epitranscriptomic modification on RNA molecules in eukaryotes. It is essential for various physiological and pathophysiological processes, including tissue development and multiple diseases [14, 15]. m^6^A is deposited by methyltransferase complex (writers), wiped off by demethylase (erasers), and recognized by binding proteins (readers) [16–18]. The asymmetric Mettl3-Mettl14 heterodimer is responsible for most m^6^A deposition on messenger RNAs (mRNAs). Mettl14 functions as a scaffold while Mettl3 is the catalytic subunit [19–22]. Homozygous Mettl3-deficient mice show embryonic lethality [23]. Similarly, deletion of Mettl14 in mouse embryos results in a significant embryonic growth delay starting from embryonic day 6.5, mainly due to differentiation resistance, further leading to embryonic death [24]. Human and mouse embryonic stem cells (ESCs) with Mettl3 knockout failed to exit naïve pluripotency and differentiate into downstream lineages [25]. m^6^A modification is indispensable for hematopoietic stem cell (HSC) specification, self-renewal, and differentiation [26–29]. Silencing Mettl3 in the adult hematopoietic system leads to blockage of HSC differentiation and aberrant accumulation of HSCs in bone marrow [30, 31]. During myelopoiesis, m^6^A was decreased, and inhibition of either Mettl3 or Mettl14 enhanced the differentiation of HSCs toward myeloid cells [28, 32]. m^6^A is also essential for T cell homeostasis and differentiation [33–35], dendritic cell maturation and activation [36], macrophage activation [37, 38] and polarization [39], and NK cell function [40].

However, the role of Mettl3-mediated m^6^A modification in B cell development and functions remains elusive. Zheng and colleagues found that *Mb1-Cre*-mediated ablation of Mettl14 resulted in the block of pro-B cell proliferation, pro-B to large pre-B, and large pre-B to small pre-B transition [41]. *Mb1-Cre*-mediated Mettl3 knockout showed a similar phenotype [42]. Since *Mb1-Cre* starts to express at the earliest pre-pro-B cell (CD19^−^B220^mid^Ig*κ*/*λ*^−^Cd43^hi^) stage [41], whether knocking out *Mettl3/Mettl14* at later stages affects B cell development and function is still unknown. Recently, Grenov et al. reported that Mettl3-mediated m^6^A modification was required for the germinal center formation and maintenance [43].

Here, we deleted Metllt3 using *Cd19-Cre* (expressing from the pro-B cell stage on [44, 45]) (*Mettl3* cKO) to see the role of Mettl3-mediated m^6^A in later stage development and function of B cells. No developmental defects of *Mettl3* cKO mice were observed. The frequency of total B cells in peripheral blood, peritoneal cavity, and liver, as well as B cell subsets at different developmental stages (pre-pro-B, pro-B, pre-B, immature B, and mature B cells) in the bone marrow, was comparable between *Mettl3* cKO mice and wild-type control (WT) littermates, consistent with previous reports by Grenov et al. [43]. Deletion of Mettl3-mediated m^6^A using *Cd19-Cre* did not affect B cell proliferation and IgG production but promoted apoptosis *in vitro*. Moreover, different stimuli (LPS, CD40L, anti-IgM, or TNF-*α*) had different effects on B cell activation. As B cell contributes to hepatic fibrosis in an antibody-independent way [46], and Mettl3 was increased in B cells from fibrotic livers in published datasets [47], we explored the function of Mettl3 on B cells in*vivo*by using CCl_4_-induced liver fibrosis model. The results showed that Mettl3 deletion in B cells does not affect liver fibrosis progression. Our study demonstrated that Mettl3 marginally affects Cd19^+^ B cell development, activation, and profibrogenic function in liver fibrosis.

## 2. Methods

### 2.1. Mice

*Mettl3^flox/flox^* mice (kindly gifted by Professor Qi Zhou [48]) were crossed with *Cd19-Cre* mice (purchased from GemPharmatech Co. Ltd, Nanjing, China) to generate *Mettl3^flox/flox^/Cd19-Cre* (*Mettl3* cKO) mice. *Mettl3^flox/flox^* littermates were used as WT controls. 6 to 8 weeks old sex- and age-matched mice were used in this study. All mice were maintained on a C57BL/6 background and housed in specific pathogen-free conditions. Animal care and experimental protocols were approved by the Institutional Animal Care and Use Committee of the Third Affiliated Hospital of Sun Yat-sen University. Primers used for genotyping were listed in Supplementary Table 1.

### 2.2. CCl_4_-Induced Liver Fibrosis

CCl_4_ (289116, Sigma-Aldrich, USA) was diluted with corn oil (O815211, Macklin, China) at a ratio of 1 : 4 and injected intraperitoneally (i.p.) into mice at 5 *μ*l/g body weight twice per week for six weeks as described previously [49]. Samples were collected 24 hours after the last CCl_4_ treatment.

### 2.3. Measurement of Liver Functions

Serum levels of liver function indicators (alanine aminotransferase (ALT), aspartate aminotransferase (AST), albumin (ALB), and alkaline phosphatase (ALP)) were detected using Hitachi 7020 automatic biochemical analyzer (Hitachi, Tokyo, Japan).

### 2.4. RNA Isolation and Quantitative Real-Time PCR (qRT-PCR)

Total RNA was extracted with TRIzol reagent (15596026, Invitrogen, USA), followed by reverse transcription with the Fast All-in-One RT Kit (RT001, ES Science, China). cDNA was used as the template in real-time PCR with SYBR Green (4707516001, Roche, Switzerland). All reactions were performed in triplicates, and *Gapdh* was used as the internal control. The relative mRNA abundance was calculated using the *ΔΔ*Ct methods. Primers were listed in Supplementary Table 2.

### 2.5. Western Blot

Cell pellets or tissues were lysed, and protein concentration was detected by the BCA method. The proteins were equally loaded to SDS-PAGE gel, transferred onto nitrocellulose membranes, and then incubated sequentially with primary and second antibodies. The protein bands were developed by Chemidoc Imaging System (Biorad®) using Immobilon ECL Ultra Western HRP Substrate (WBULS500, Millipore, USA). The antibodies used were listed in Supplementary Table 3.

### 2.6. H&E, PSR, and Immunohistochemistry Staining

Mouse livers were perfused with ice-cold PBS, fixed with 4% phosphate-buffered formalin, embedded in paraffin, and cut into sections. Sections were stained for picrosirius red (PSR) or hematoxylin and eosin (H&E) using standard procedures. For *α*SMA immunohistochemistry staining, liver slices were dewaxed, rehydrated, and then incubated with an anti-*α*SMA antibody (ab5694, Abcam, England) overnight at 4°C, followed by a secondary antibody. The color was developed by incubation with a Dako RealTM kit (K5007, Dako, Denmark) and scanned under the microscope (Nikon, Japan). Quantification for the positive areas of PSR and *α*SMA was analyzed by 5 random fields (100×) for each individual.

### 2.7. Purification of Splenic B Cells

Splenic B cells were purified with EasySep™ Mouse CD19 Positive Selection Kit II (18954, STEMCELL, Canada) on the EasyEights™ EasySep™ Magnet (18103, STEMCELL, Canada) according to the manufacturer's instructions.

### 2.8. Liver Lymphocyte Isolation

The liver was cannulated with a 25-gauge needle through the portal vein and perfused with 10 ml of ice-cold PBS. After removing the gall bladder, the liver was cut into segments and digested with 0.02% collagenase IV (C5138, Sigma-Aldrich, USA, 5 ml per liver) for 45 mins at 37°C on a shaker at the speed of 70 rpm. The liver slurry was centrifuged for 3 mins at 30 g. The supernatants were passed through a 70 *μ*m mesh cell strainer (352350, BD Falcon, USA) and then centrifuged for 10 mins at 300 g at 4°C. The cell pellets were resuspended in 5 ml of mouse 1 × lymphocyte separation medium (7211011, DAKEWEI, China), overlaid by 0.5 ml RPMI-1640, then centrifuged at 800 g for 30 minutes at 4°C with no brakes. Lymphocytes at the interface were harvested, washed with RPMI-1640 supplemented with 5% fetal bovine serum (FBS, FSP500, ExCell, China), and used for further analyses.

### 2.9. Isolation of Lymphocytes from Peritoneal Cavity, Spleen, Blood, and Bone Marrow

Mice were anesthetized and exposed abdominal cavity. Sterilized PBS was used to wash the peritoneal cavity, and then the peritoneal lavage fluid was collected and centrifuged at 300 g for 5 mins at 4°C. Cells obtained were used for further analysis. Spleens were minced through a nylon mesh (Cell Strainer, 352340, BD Falcon, USA) to obtain single-cell suspensions in RPMI-1640 containing 5% FBS. Erythrocytes were lysed by incubating in RBC lysis buffer (140 mM NH4Cl, 17 mM Tris-HCl, and pH 7.65) for 3 minutes on ice. Peripheral blood was collected in EDTA-containing tubes, then underlaid with Ficoll-Paque™ PLUS (17-1440-02, GE Healthcare, USA), and centrifuged at 1000 g at room temperature for 20 minutes with no brakes. Lymphocytes were collected from the interface. Femur and tibia bones were used to isolate bone marrow-derived lymphocytes. Both ends of the bone were carefully cut with sharp dissecting scissors. Bone marrow cells were flushed using PBS and then centrifuged at 300 g for 5 mins at 4°C. The cell pellets were resuspended in 1 ml RBC lysis buffer and lysed for 3 mins on ice. 5 volume of PBS was added to stop the reaction. Lymphocytes were collected by centrifugation at 300 g at 4°C, washed twice with RPMI-1640 supplemented with 5% FBS, and used for further analyses.

### 2.10. Flow Cytometry

Flow cytometric analysis was performed on BD® LSR II Flow Cytometer (Marshallscientific, USA), and data were analyzed with FlowJo10.0 software (Treestar, Ashland, OR, USA). Anti- mouse B220-FITC (103205, Biolegend, USA), CD43-PE-Cy7 (143210, Biolegend, USA), CD24-PE (101807, Biolegend, USA), CD19-PE-Cy7 (552854, BD, USA), CD19-BV421 (115520, Biolegend, USA), CD19-PE (115508, Biolegend, USA), CD69-FITC (104506, Biolegend, USA), CD5-PE (100608, Biolegend, USA), CD86-APC (105011, Biolegend, USA), CD95-PE (152608, Biolegend, USA), BP-1-Alexa Fluor 647 (108312, Biolegend, USA), IgD-APC (405714, Biolegend, USA), IgM-PE (406507, Biolegend, USA), and corresponding isotype control antibodies were purchased from Biolegend. Antibodies were listed in Supplementary Table 3.

### 2.11. Proliferation and Activation Analysis of Isolated B Cells

Carboxyfluorescein succinimidyl ester (CFSE, C34554, Invitrogen, USA) was dissolved at 5 mM in DMSO and stored at -80°C. The isolated B cells were washed twice with RPMI-1640, resuspended at 5 × 10^7^ cells/ml in warm RPMI-1640 containing a 5 *μ*M CFSE, incubated for 10 minutes at 37°C in the dark, washed 3 times with RPMI 1640 containing 5% FBS, and resuspended in RPMI-1640 supplemented with 10% FBS. 2 × 10^5^ cells in 100 *μ*l RPMI-1640 containing 10% FBS were plated into a flat-bottom 96-well plate well. Another 100 *μ*l RPMI-1640 containing 10% FBS and stimulating reagents (anti-IgM, CD40L, LPS, or TNF-*α*) was added. Working concentration for each stimulating reagent for activation and apoptosis analysis was as follows: anti-IgM (affinipure F(ab')_2_-fragment goat antimouse IgM, *μ* chain specific, 1 *μ*g/ml; 115-005-006, Jackson ImmunoResearch, USA), CD40L (100 ng/ml; 34-8512-80, eBioscience, USA), LPS (2 *μ*g/ml; L2880, Sigma-Aldrich, USA), and TNF-*α* (50 ng/ml; 315-01A, Peprotech, USA). After 2 days, cells were used for flow cytometry analysis, and supernatants were collected and analyzed for IgG levels by Mouse IgG Total Uncoated ELISA kit (88-50400-22, Invitrogen, USA) according to the manufacturer's instructions. Working concentration for each stimulating reagent for proliferation analysis was as follows: anti-IgM (2 *μ*g/ml), CD40L (200 ng/ml), LPS (5 *μ*g/ml), and TNF-*α* (50 ng/ml). After 5 days, cells were collected and used for flow cytometry analysis.

## 3. Results

### 3.1. Generation of *Cd19-Cre*-Mediated B Cell-Specific *Mettl3* Knockout Mice

**Figure 1:**
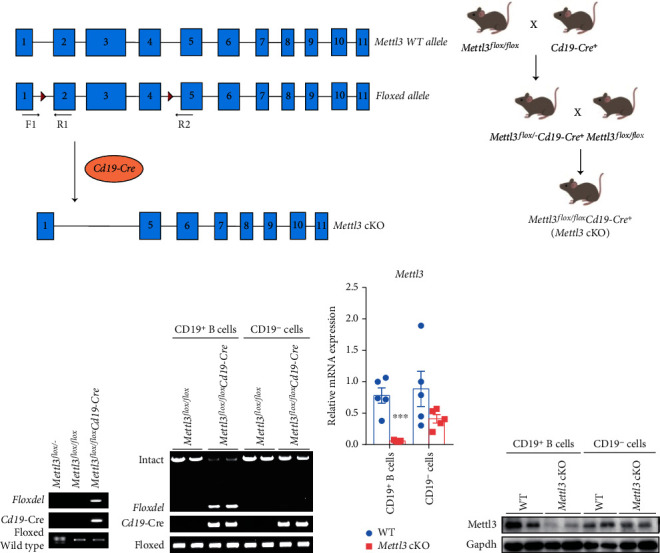
Construction and characterization of B cell-specific *Mettl3* knockout mice using *Cd19-Cre.* (a) Strategy of *Cd19-Cre*-mediated *Mettl3* knockout mouse construction. Floxed-Mettl3 allele was generated by flanking exons 2 and 4 with loxP sites. B cell-specific *Mettl3* knockout mice (*Mettl3* cKO) were obtained by crossing floxed-Mettl3 mice with *Cd19-Cre* transgenic mice. (b) Schematic diagram of breeding strategies of *Mettl3* cKO mice. (c) Genomic PCR for the tail of indicated genotype. The top lane (floxdel) showed the exon 2-4 deleted alleles (amplified using Mettl3-F1 and Mettl3-R2 primer shown in (a)). The middle lane (*Cd19-Cre*) showed the effective insertion of *Cd19* promoter-driven *Cre*. The bottom lane displayed genotyping of heterozygous (*Mettl3^flox/-^*) or homozygous (*Mettl3^flox/flox^*) flox flanking alleles (amplified by Mettl3-F1 and Mettl3-R1 primer shown in (a)). (d) Genomic PCR analysis for CD19^+^ B cells and CD19^−^ cells isolated from the spleen of mice with indicated genotype. Intact (upper) and floxdel (lower, with the exon 2-4 deleted alleles) lanes were amplified using Mettl3-F1 and Mettl3-R2 primer shown in (a). (e) qRT-PCR for *Mettl3* of CD19^+^ B cells and CD19^−^ cells isolated from WT and *Mettl3* cKO mouse spleen. (f) Western blot for Mettl3 of CD19^+^ B cells and CD19^−^ cells isolated from WT and *Mettl3* cKO mouse spleen. Data in (e) were presented as means ± SEM with the indicated significance (∗∗∗*P* < 0.001; student's *t*-test).

By knocking out Mettl3 or Mettl14 using *Mb1-Cre* (starts to express at the earliest pre-pro-B cells), previous studies showed that m^6^A plays an essential role in early B cell development [41, 42]. To investigate the role of m^6^A modification in B lymphocyte development and function at later stages, we generated Mettl3 conditional knockout mice (*Mettl3* cKO) by crossing *Mettl3^flox/flox^* mice (with loxP sites flanking exons 2 and 4) with *Cd19*-*Cre* mice (placing Cre recombinase under the control of the endogenous Cd19 promoter/enhancer elements by inserting Cre recombinase gene linked by the P2A self-cleaving peptide before translation stop codon of the Cd19 gene, without disrupting endogenous Cd19 expression and function) which starts to express Cre from the pro-B stage on (Figures 1(a) and 1(b)) [44, 45]. Genotype was monitored by genomic PCR of mouse tails (Figure 1(c)). To further confirm B cell-specific Mettl3 knockout, we sorted splenic CD19^+^ B cells and CD19^−^ cells from WT and *Mettl3 cKO* mice and conducted genomic PCR, western blot, and RT-qPCR for Mettl3 (Figures 1(d)–1(f)). The results showed specific and efficient knockout of Mettl3 occurred only in CD19^+^ B cells.

### 3.2. Loss of *Mettl3* in CD19^+^ Cells Has Minimal Effects on B Cell Distribution in Peripheral

**Figure 2:**
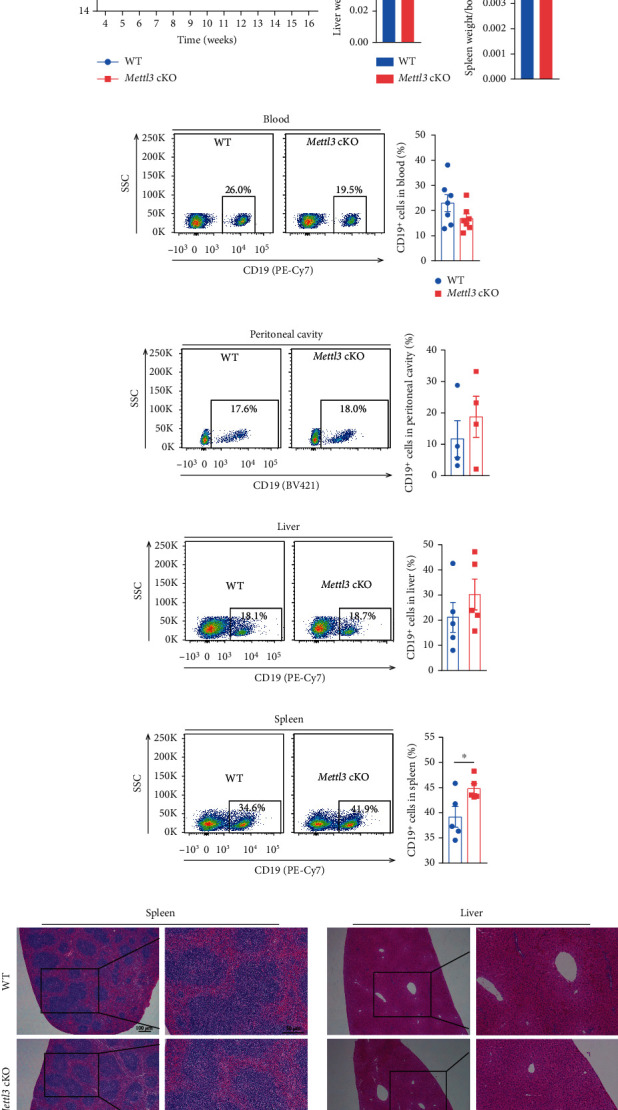
Loss of *Mettl3* in Cd19^+^ cells has minimal effects on mouse development and B cell distribution in peripheral. (a) Body weight of control and *Mettl3* cKO littermates at different time points after birth (*n* = 24/group). (b) The ratio of liver weight to body weight for mice in indicated groups (*n* = 24/group). (c) The ratio of spleen weight to body weight for mice in indicated groups (*n* = 24/group). (d)–(g) Representative flow cytometry plots (left) and quantification (right) for B cell marker CD19 of lymphocytes isolated from peripheral blood (d) (*n* = 7/group), peritoneal cavity (e) (*n* = 4/group), liver (f) (*n* = 5/group), and spleen (g) (*n* = 5/group) at indicated groups. (h) Representative photographs of HE staining of the spleen (left) and liver (right) of WT and *Mettl3* cKO mice. Scale bar = 100 *μ*m and 50 *μ*m as indicated. Data in (c and g) were presented as means ± SEM with the indicated significance (∗*P* < 0.05; student's *t*-test).

We first explored whether knocking out Mettl3 in B cells affects mouse development. The body weight, liver weight, and the ratio of liver weight to body weight were indistinguishable between *Mettl3* cKO mice and *Mettl3^flox/flox^* control littermates (Figures 2(a) & 2(b) and Supplementary Figure 1A & 1B). Mice were born at expected Mendelian frequency, and no infection or other discernable differences were observed during regular feeding (data not shown). Interestingly, the spleen weight and the ratio of spleen weight to body weight were slightly increased in *Mettl3* cKO groups (Figure 2(c) and Supplementary Figure 1C). Deletion of Mettl3 or Mettl14 with *Mb1-Cre* resulted in a significant decrease of B cells in the peripheral and even disappeared in the spleen and peritoneal cavity [41, 42]. We determined B cell percentage in peripheral blood, peritoneal cavity, liver, and spleen and found that there was no significant difference in CD19^+^ B cell fraction between WT and *Mettl3* cKO mice in peripheral blood, peritoneal cavity, and liver (Figures 2(d)–2(f) and Supplementary Figures 2A–2D). However, the proportion of CD19^+^ B cells in the spleen was slightly but significantly increased in *Mettl3* cKO individuals (Figure 2(g)), which may contribute to the increased spleen weight of *Mettl3* cKO mice (Figure 2(c) and Supplementary Figure 1C). Histological analysis with H&E staining also showed no structural and histological abnormalities in the spleen and liver of *Mettl3* cKO mice compared to WT controls (Figure 2(h)). These results indicated that knocking out Mettl3 in B cells using *Cd19-Cre* minimally affects B cell development.

### 3.3. Depletion of *Mettl3* in CD19^+^ Cells Does Not Affect B Cell Development and Maturation in Mice

**Figure 3:**
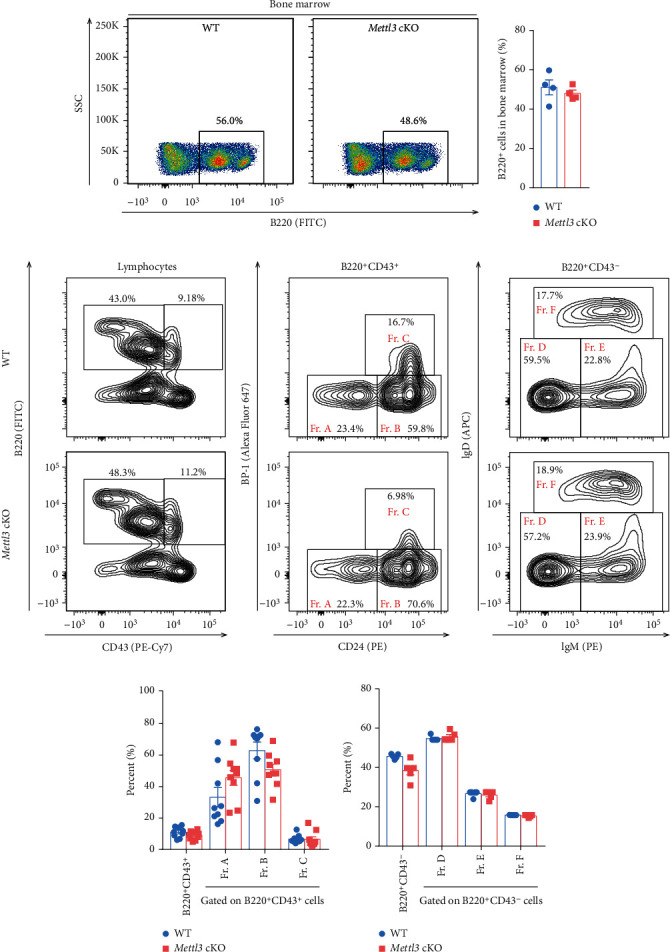
Loss of *Mettl3* does not affect B cell development and maturation in bone marrow. (a, b) Representative flow cytometry plots (a) and quantification (b) of B cells in the bone marrow of indicated groups (*n* = 4/group). (c)–(e) Representative flow cytometry plots of B cell subpopulations in the bone marrow of WT and *Mettl3* cKO mice. B220^+^CD43^+^ lymphocytes were further analyzed for fraction Fr. A (CD24^−^ BP1^−^), Fr. B (CD24^+^BP1^−^), and Fr. C (CD24^+^BP1^+^), and B220^+^CD43^−^ lymphocytes were further analyzed for fraction Fr. D (IgM^−^IgD^−^), Fr. E (IgM^+^IgD^−^), and Fr. F (IgM^+^IgD^+^). (d, e) Quantification of subpopulations in B220^+^CD43^+^ lymphocytes (d) (*n* = 9/group) and B220^+^CD43^−^ lymphocytes (e) (*n* = 5/group). Data in (b, d, and e) were presented as means ± SEM.

To explore the role of Mettl3 in B cell development, we analyzed the proportion of B cells at different developmental stages in the bone marrow. The percentage of B220^+^ B cells in the bone marrow was not affected in *Mettl3* cKO mice compared to WT controls (Figures 3(a) & 3(b) and Supplementary Figure 2E). Whole bone marrow cells were segregated based on B220 and CD43 expression (Figure 3(c), left) and divided B cell precursors into B220^+^CD43^+^ progenitor cells based on CD24 and BP-1 expression (Figure 3(c), center) and more mature B220^+^CD43^−^ populations based on surface IgM and IgD expression (Figure 3(c), right) [50, 51]. Both CD43^+^ populations (containing the most immature B-cell populations in the marrow) and CD43^−^ populations (mainly containing pre-B-cells, immature and mature B cells) were comparable between WT and *Mettl3* cKO littermates (Figures 3(d) and 3(e)). Moreover, there is no significant difference in the proportion of pre-pro-B (fraction (Fr.) A, B220^+^CD43^+^CD24^–^), early pro-B (Fr. B, B220^+^CD43^+^CD24^+^BP1^–^), and late pro-B (Fr. C, B220^+^CD43^+^CD24^+^BP1^+^) fractions in the bone marrow between *Mettl3* cKO mice and WT controls (Figure 3(d)). We also observed a minimal difference of IgM^+^IgD^−^ immature B cells and IgM^+^IgD^+^ mature B cells in B220^+^CD43^−^ fractions (Figure 3(e)). Therefore, the knockout of Mettl3 in B cells with *Cd19-Cre* barely influenced B cell development and maturation.

### 3.4. Loss of *Mettl3* Has Minimal Effects on B Cell Activation and Proliferation but Promotes Apoptosis upon Stimulation *In Vitro*

**Figure 4:**
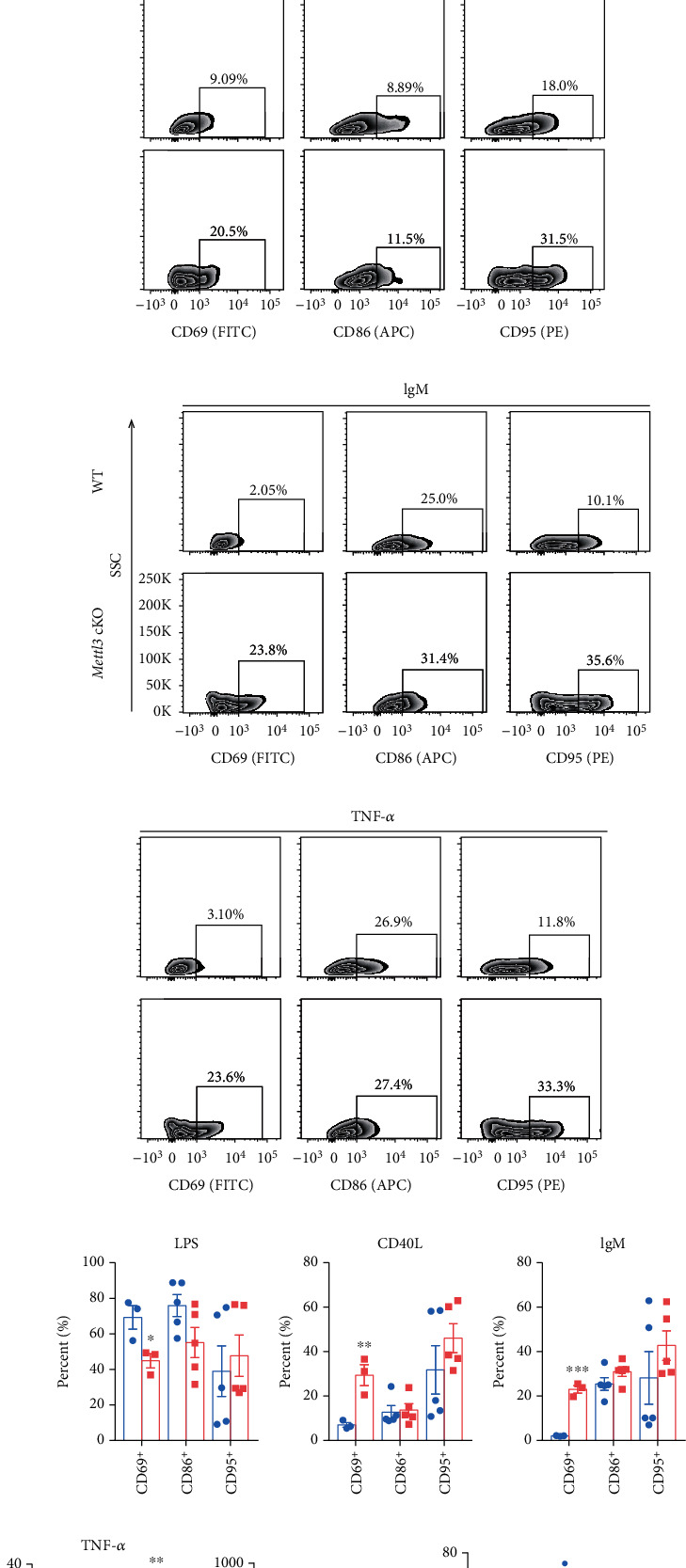
Knockout of *Mettl3* minimally affects B cell activation upon stimulation *in vitro.* B cells were isolated from the spleen of WT and *Mettl3* cKO mice by using CD19 microbeads and treated with LPS (2 *μ*g/ml), CD40L (100 ng/ml), anti-IgM (1 *μ*g/ml), or TNFa (50 ng/ml). Cells were cultured for 2 days, then collected and subjected to flow cytometry. (a)–(h) Representative flow cytometry plots (a)–(d) and quantification (e)–(h) of indicated populations in indicated groups. (i) ELISA assay for IgG secretion of B cells treated with LPS (2 *μ*g/ml), CD40L (100 ng/ml), and anti-IgM (1 *μ*g/ml) for 2 days. (j) RT-qPCR analysis for indicated genes in B cells of indicated groups upon LPS stimulation (*n* = 3/group). Data in (e)–(h) were presented as means ± SEM with the indicated significance (∗*P* < 0.05, ∗∗*P* < 0.01, ∗∗∗*P* < 0.001; student's *t*-test).

**Figure 5:**
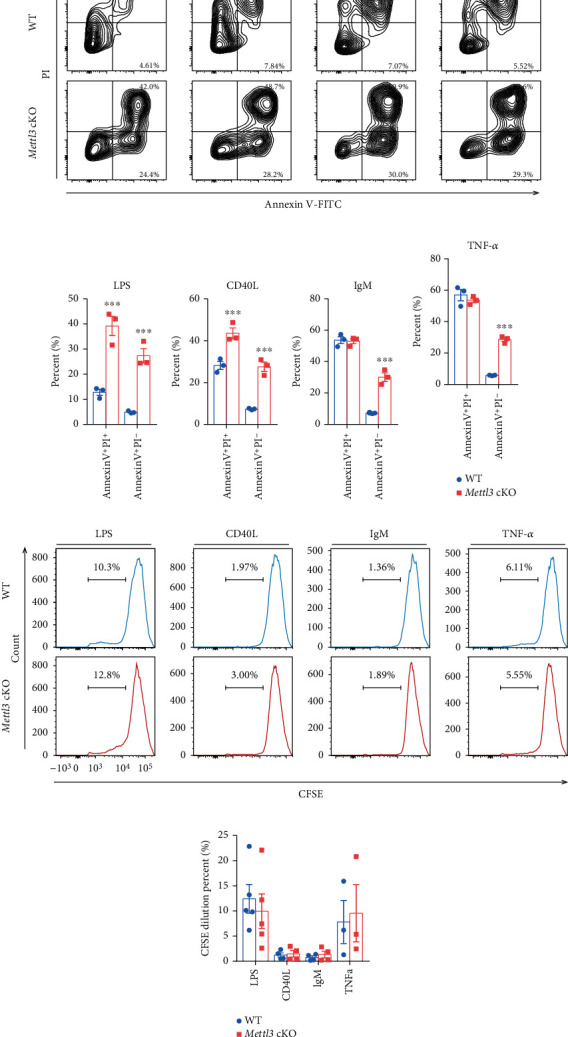
Knockout of *Mettl3* promotes B cell apoptosis, but barely affects proliferation. B cells were isolated from the spleen of WT and *Mettl3* cKO mice using CD19 microbeads and treated with LPS (2 *μ*g/ml), CD40L (200 ng/ml), anti-IgM (1 *μ*g/ml), or TNFa (50 ng/ml). Cells were cultured for 2 days, then collected and subjected to flow cytometry. (a)–(e) Representative flow cytometry plots (a) and quantification (b)–(e) of indicated populations in indicated groups. (f, g) B cells were isolated from the spleen of WT and *Mettl3* cKO mice, labeled with CFSE, treated with LPS (5 *μ*g/ml), CD40L (200 ng/ml), anti-IgM (2 *μ*g/ml), or TNFa (50 ng/ml) for 5 days, then collected and subjected to flow cytometry. Representative flow cytometry plots (f) and quantification (g) for cytometry analysis for the proliferation of CD19^+^ splenic B cells in indicated groups. Data in (b)–(e) and (g) were presented as means ± SEM with the indicated significance (∗∗∗*P* < 0.001; student's *t*-test).

To investigate whether Mettl3 regulates B cell function, we isolated B cells from the spleen of WT and *Mettl3* cKO mice and incubated them with LPS (2 *μ*g/ml), the ligand for CD40 (CD40L, 100 ng/ml), anti-IgM (1 *μ*g/ml), or TNF-*α* (50 ng/ml) for 2 days. B cell activation was assessed by flow cytometry based on the expression level of CD69, CD86, and CD95. CD86 and CD95 showed that B cells from WT and *Mettl3* cKO mice were activated at the same degree upon LPS, CD40L, and anti-IgM stimulation (Figures 4(a)–4(d) and Supplementary Figure 1D). However, the expression of CD69 showed a different pattern. B cells from *Mettl3* cKO mice expressed higher activation marker CD69 in response to CD40L, anti-IgM, or TNF-*α*, while decreased in response to LPS (Figures 4(e)–4(h)). Besides, TNF-*α* also induced a higher CD95 expression level in *Mettl3* cKO B cells (Figure 4(h)). However, IgG levels in the culture medium were comparable between WT and *Mettl3* KO B cells upon LPS, CD40L, and anti-IgM stimulation (Figure 4(i)). The RT-qPCR analysis also showed consistent mRNA level of B cell survival factor *Tnfsf13b* (encoding BAFF), cytokines (*Ltβ*, *Il10*, and *Tgfβ1*), and chemokines (*Cxcl12*, *Cxcl13*, and *Ccl21*) in LPS-activated B cells from WT and *Mettl3* cKO mice (Figure 4(j)). Next, we monitored the effects of *Mettl3* knockout on B cell apoptosis under different treatments (Figure 5(a)). The proportion of early apoptotic cells (Annexin^+^PI^−^) was increased in *Mettl3*cKO B cells upon LPS and CD40L stimulation, while the late apoptotic cells (Annexin^+^PI^+^) were more common in *Mettl3*cKO B cells with different stimuli (Figures 5(b)–5(e)). In addition, we analyzed B cell proliferation and observed that there was comparable residual CFSE fluorescence intensity of B cells from WT and *Mettl3* cKO mice, indicating that deletion of Mettl3 has little influence on B cell proliferation (Figures 5(f) & 5(g) and Supplementary Figure 1E). These results showed that Cd19-mediated B cell-specific Mettl3 knockout had minimal impact on B cell proliferation and IgG production but could promote apoptosis, and the effects on B cell activation varies from different stimuli.

### 3.5. Mettl3 Is Dispensable for the Profibrogenic Activity of B Cells in Liver Fibrosis

**Figure 6:**
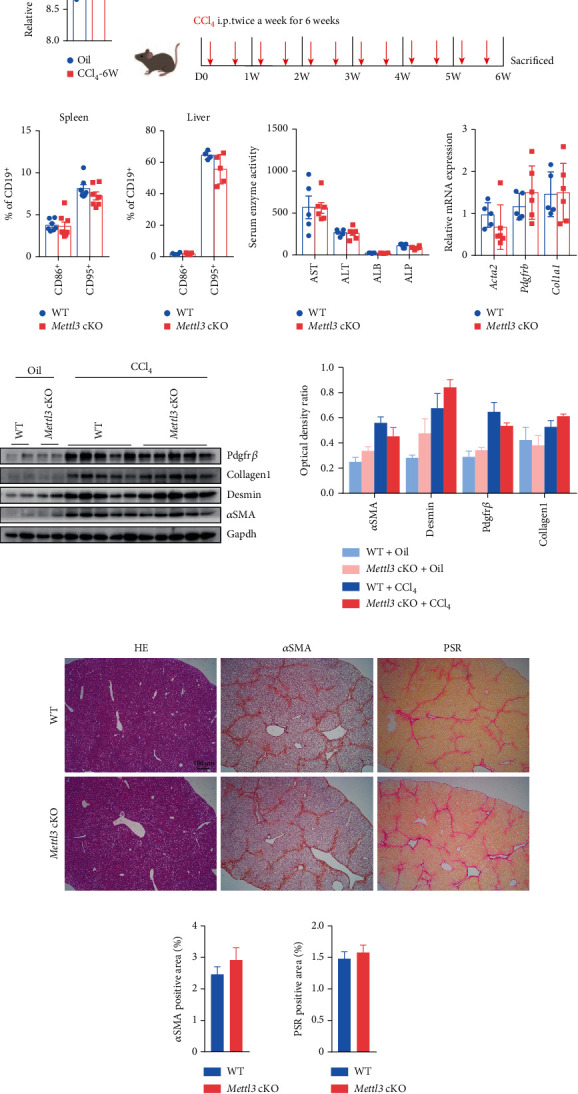
Knockout of *Mettl3* does not affect the pro-fibrogenic activity of B cells in liver fibrosis. (a) Quantitation of mRNA expression level of *Mettl3* in B cells isolated from mouse livers treated with CCl_4_ or Oil (control) for 6 weeks. Results were retrieved from published microarray data [47]. (b) Schematic diagram of CCl_4_-induced mouse liver fibrosis. (c, d) Quantification of flow cytometry analysis for B cell activation marker CD86 and CD95 in the spleen (c) and liver tissues (d) of WT control and *Mettl3* cKO mice after fibrosis induction. (e) Serum levels of alanine aminotransferase (ALT), aspartate aminotransferase (AST), albumin (ALB), and alkaline phosphatase (ALP) levels in indicated groups 24 h after the last CCl_4_ treatment (*n* = 5 for WT control and *n* = 6 mice for *Mettl3* cKO). (f) RT-qPCR for profibrogenic genes of liver tissues in indicated groups (*n* = 5 for WT control and *n* = 6 for *Mettl3* cKO). (g) Representative western blot (left) and quantification (right) for profibrogenic genes in indicated groups (*n* = 5/group). Gapdh was used as a loading control. (h) Representative photographs of H&E staining (left), *α*SMA immunohistochemistry staining (middle), and picrosirius red (PSR) staining (right) of fibrotic liver sections from WT control and *Mettl3* cKO mice. (i, j) Quantification of the aSMA positive area (i) and PSR staining positive area (j) in indicated groups (*n* = 5 for WT control and *n* = 6 for *Mettl3* cKO). Data in (a), (c)–(f), and (i, j) were presented as means ± SEM with the indicated significance (∗*P* < 0.05; student's *t*-test).

The above results showed that deletion of Mettl3 in B cells does not affect B cell specification but seems comprehensively affect B cell activation *in vitro*. B cell activation and function *in vivo* result from the integration of multiple signals and are much more complicated than *in vitro*. B cells can promote hepatic fibrosis progression [47, 52]. However, the mechanisms that regulate B cell activation and function during liver fibrosis were not fully understood. Given the published dataset, we found that Mettl3 was upregulated in B cells of fibrotic livers [47] (Figure 6(a)), indicating that Mettl3 was involved in B cell function in liver fibrosis. To identify whether Mettl3 regulates B cell function *in vivo*, we subjected WT and *Mettl3* cKO mice to CCl_4_-induced liver fibrosis (Figure 6(b)), a well-established and widely-used hepatotoxic fibrosis model [53]. CCl_4_-induced liver fibrosis could reproduce pathological features of chronic liver diseases caused by various etiologies. This model also avoids activation of a specific subset of lymphocytes that occurred in LPS or Concanavalin A-induced liver damage [54, 55]. 24 hours after the last CCl_4_ injection, flow cytometry assay for B cell activation in the spleen and liver was conducted. The results showed that B cell activation in *Mettl3* cKO mice was the same as in WT controls, both in the spleen and liver (Figures 6(c) and 6(d)). Serum indicators of liver function showed no discernible difference between WT and *Mettl3* cKO mice (Figure 6(e)). Liver fibrosis between WT and *Mettl3* cKO mice was also comparable, evidenced by RT-qPCR (Figure 6(f)) and western blot (Figure 6(g)) for profibrotic markers (*Acta2* (encoding *α*-smooth muscle actin (*α*SMA)), *Col1a1* (encoding collagen type I), and *Pdgfrb* (encoding Pdgfr*β*)), H&E staining, PSR staining, and immunohistochemical staining for *α*SMA of mouse liver tissues (Figures 6(h)–6(j)). These results indicated that knocking out Mettl3 in B cells using *Cd19-Cre* does not affect the profibrogenic activity of B cells in liver fibrosis.

## 4. Discussion

Over the past decades, numerous studies have addressed that epigenetic modifications control various aspects of B cell development [56]. For instance, deficiency in BMI1 or MEL18 leads to a block of B cell development [57, 58]. EZH2 or MYSM1 orchestrates early B cell development [59, 60]. Loss of HADC1, HADC2, or HADC7 also impairs early B cell development [61, 62].

m^6^A is the most frequent chemical modification of mRNA and lncRNA in eukaryotes. It controls multiple physiological and pathophysiological processes [14, 15]. During B cell development, inhibition of Mettl3-mediated m^6^A modification in HSCs resulted in a block of HSC differentiation and subsequent decreased B cell frequency in the peripheral [30, 31]. Deleting *Mettl3* or *Mettl14* in the very early stage of B cell specification using *Mb1-Cre* resulted in blockage of B cell differentiation in general, particularly pro-B to large pre-B and large pre-B to small pre-B transitions [41, 42]. Since *Cd19*-Cre^+^ mice express Cre from the pro-B cell stage on [44, 45], later than *Mb1*-Cre^+^ mice, we generated *Cd19-Cre*-mediated *Mettl3* cKO mice to investigate the effect of m^6^A modification at later stages of B cell development and function. We observed that the ratio of total B cells in the *Mettl3* cKO mice's peripheral blood, peritoneal cavity, and liver was equivalent to that in the WT controls. However, there is a slight increase in splenic B cell proportion in *Mettl3* cKO mice. The percentage of pre-pro-B, pro-B, pre-B, immature, and mature B cells in the bone marrow of WT and *Mettl3* cKO mice is also comparable, indicating that loss of Mettl3 from the pro-B stage on minimally affects B cell development. These results suggested that the requirement of Mettl3-mediated m^6^A during differentiation is stage-specific, and earlier stage deficiency may bring more severe outcomes.

B cells are an important part of the adaptive immune system by presenting antigens, secreting cytokines, and producing antibodies. B cells were mainly activated in T cell-dependent and T cell-independent manners *in vivo* [63, 64]. For *in vitro* experiments, various stimulants, including LPS, sCD40L, anti-IgM, TNF-a, IFN-*γ*, and CpG ODNs, were used to trigger B cell activation. CD40L/CD40 ligand-receptor pair provides signals to B cells and induces T cell-dependent proliferation, survival, immunoglobulin class switching, antibody secretion, and germinal center formation [65]. LPS is also a potent stimulant of B cells and induces B cells to proliferate, produce antibodies, and secrete IL-6 through TLR4 [66–68]. Crosslinking the BCR with anti-IgM as a surrogate antigen induces T cell-independent B cell activation and moderate B cell proliferation [69, 70]. TNF-*α* has been reported to be related to B cell proliferation, apoptosis, and the expression of individual molecules on the membrane, including CD19 and CD45 [71–73]. Thus, we isolated splenic B cells and treated them with LPS, CD40L, anti-IgM, or TNF-*α* to investigate whether depletion of *Mettl3* in B cells could affect B cell activation, proliferation, and apoptosis *in vitro*. Our results indicated that loss of *Mettl3* resulted in increased apoptosis with no difference in the proliferation and IgG production upon different treatments. *Cd19-Cre*-mediated Mettl3 deletion makes B cells prone to apoptosis upon stimulation. However, different stimuli had different effects on B cell activation marker expression *in vitro*, consistent with previous studies that different stimulations may activate different subpopulations of B cells and result in different B cell phenotypes [74–76]. To see the integrated effect of different stimulation on Mettl3 cKO B cells, we used the CCl_4_-induced liver fibrosis model, in which Mettl3 in B cells was upregulated. We observed that *Cd19-Cre*-mediated Mettl3 deletion does not affect the profibrogenic activity of B cells in CCl_4_-induced liver fibrosis *in vivo*.

B cells are traditionally known for producing antibodies and mediating humoral immune responses. However, recent studies showed that B cells are critical modulators of adaptive and innate immune responses [8]. Liver fibrosis is a dynamic wound-healing process characterized by the accumulation of extracellular matrix [77]. The role of B cells in liver fibrosis has been extensively explored recently [47, 52]. Increased B cells in the fibrotic liver can exacerbate liver fibrosis in an antibody-independent manner by producing proinflammatory mediators to stimulate the hepatic stellate cells, a key driver of liver fibrosis [46]. Activated hepatic stellate cells produce retinoic acid, inducing B cell survival, plasma cell maturation, and IgG secretion [47]. The profibrotic role of B cells in the CCl_4_-induced liver fibrosis model depends on the myeloid differentiation primary response 88 (MYD88), which is indispensable for proper activation and proinflammatory cytokine production [47]. Here, we showed that the profibrogenic activity of B cells in liver fibrosis is independent of cell-autonomous Mettl3-mediated m^6^A modification.

During the preparation of this manuscript, another study that created *Mettl3^flox/flox^Cd19-Cre* mice was published and showed consistent results with our research: no obvious developmental defects of B cells in *Mettl3^flox/flox^Cd19-Cre* mice were observed. However, they found that Mettl3-mediated m^6^A is essential for B cell survival and proliferation in the germinal center [43]. Furthermore, another study showed that m^6^A is vital for class switch recombination during the maturation of B cells [42]. In addition to our observation that B cell activation and apoptosis from Mettl3 knockout mice were differentially affected by different stimulants treatment *in vitro*, further exploration of Mettl3 on other aspects of B cell immunity with different models was worth further investigation.

## 5. Conclusion

Our work showed that Mettl3-mediated m^6^A is not required for B cell development, proliferation, and the profibrogenic function of B cells in liver fibrosis when deleted from the pro-B stage on using *Cd19-Cre*, strengthening the idea that B cell development and function are delicately controlled at different stages and contexts.

## Data Availability

The experiment data used to support the findings of this study are included in the article.

## Supplementary Materials

Supplementary Materials Supplementary Figure 1: loss of Mettl3 in CD19^+^ cells has minimal effects on B cell activation and proliferation. Supplementary Figure 2: gating strategy for B cells from different tissues. (1) Primers for genotyping were listed in supplementary Table 1. (2) Primers for RT-qPCR were listed in supplementary Table 2. (3) Antibodies used for western blot were listed in supplementary Table 3. (4) Reagents.
