# Supplementary material for: Deletion of Mettl3 at the Pro-B Stage Marginally Affects B Cell Development and Profibrogenic Activity of B Cells in Liver Fibrosis
Source: J Immunol Res. 2022 Jun 14;2022:8118577. doi: 10.1155/2022/8118577 (PMC9213183; doi:10.1155/2022/8118577)
Supplement: Supplementary Materials — Supplementary Figure 1: loss of Mettl3 in CD19+ cells has minimal effects on B cell activation and proliferation. Supplementary Figure 2: gating strategy for B cells from different tissues. (1) Primers for genotyping were listed in supplementary Table 1. (2) Primers for RT-qPCR were listed in supplementary Table 2. (3) Antibodies used for western blot were listed in supplementary Table 3. (4) Reagents. [file 8118577.f1.docx]

**Deletion of Mettl3 at the pro-B stage marginally affects B cell development and pro-fibrogenic activity of B cells in liver fibrosis**

Xinmei Kang^1, #^, Shuhan Chen^1, #^, Lijie Pan^2^, Xiaoqi Liang^2^, Di Lu^1^, Huaxin Chen^1^, Yanli Li^1^, Chang Liu^1^, Mian Ge^4^, Qi Zhang^1, 2, 3, *^, Qiuli Liu^1, *^, Yan Xu^1, *^

1 Biotherapy Center, The Third Affiliated Hospital, Sun Yat-sen University, Guangzhou, China

2 Cell-gene Therapy Translational Medicine Research Center, The Third Affiliated Hospital, Sun Yat-sen University, Guangzhou, China

3 Guangdong Provincial Key Laboratory of Liver Disease Research, The Third Affiliated Hospital, Sun Yat-sen University, Guangzhou, China

4 Department of Anesthesiology, the Third Affiliated Hospital, Sun Yat-sen University, Guangzhou, China

*Correspondence: Dr. Yan Xu, Biotherapy Centre, the Third Affiliated Hospital of Sun Yat-sen University, 600# Tianhe Road, Guangzhou 510630, China. Tel.: 86-20-82179071, Fax: 86-20-85253305, E-mail: xuyan55@mail.sysu.edu.cn; Dr. Qiuli Liu, Biotherapy Centre, the Third Affiliated Hospital of Sun Yat-sen University, 600# Tianhe Road, Guangzhou 510630, China. Tel.: 86-20-85268037, Fax: 86-20-85253305, E-mail: liuqli3@mail.sysu.edu.cn; Dr. Qi Zhang, Biotherapy Centre, the Third Affiliated Hospital of Sun Yat-sen University, 600# Tianhe Road, Guangzhou 510630, China. Phone: 86-20-85253106, Fax: 86-20-85253305, E-mail: zhangq27@mail.sysu.edu.cn;

#Xinmei Kang and Shuhan Chen contributed equally to this work.

**Supplementary materials**

**Supplementary Figure.1**

**
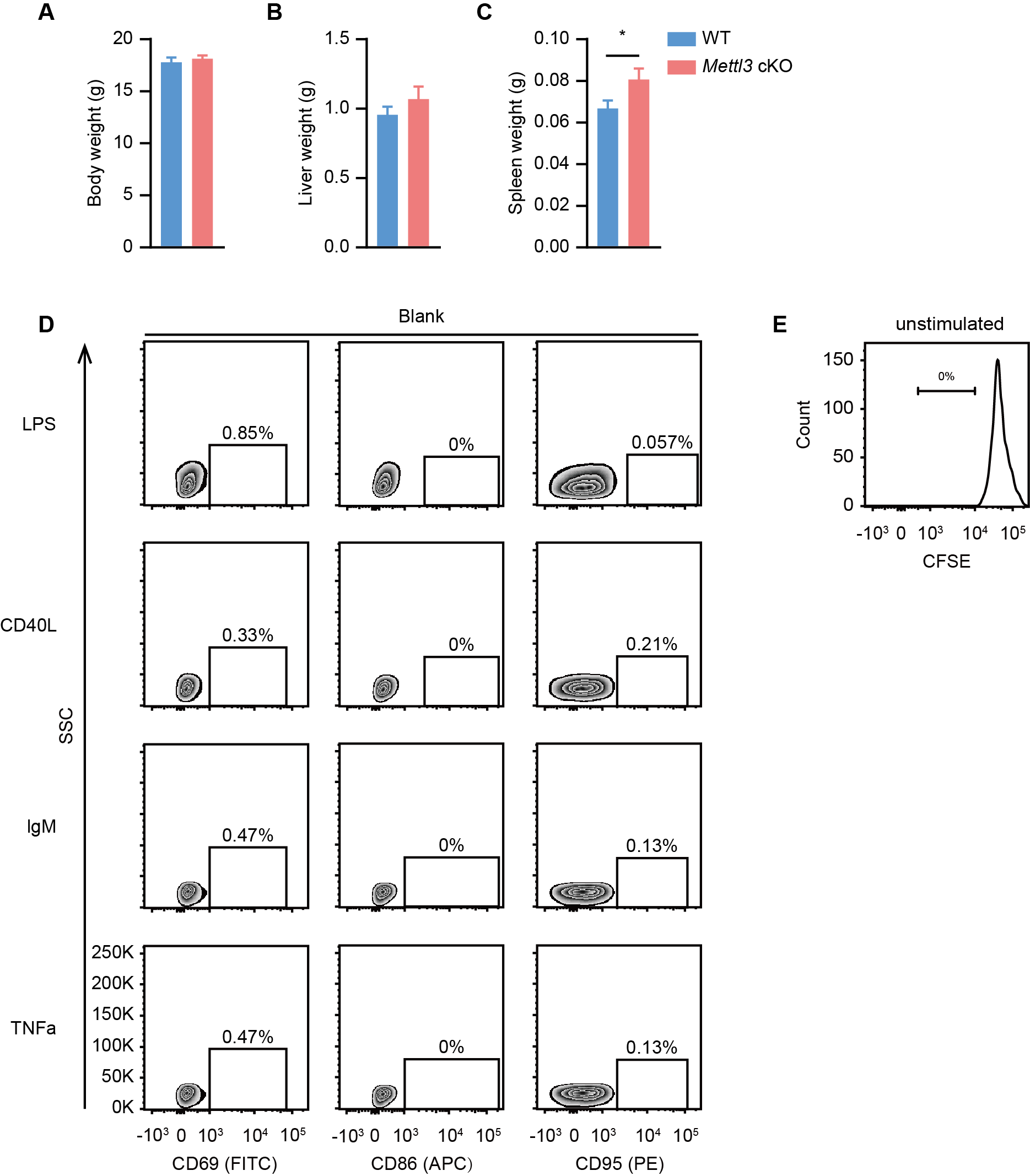
**

**Supplementary Figure.1. Loss of Mettl3 in CD19^+^ cells has minimal effects on B cell activation and proliferation. Related to Figure. 2, Figure. 4, and Figure. 5.**

(A) Body weight of WT and *Mettl3* cKO littermates (n = 24/group). (B) Liver weight of WT and *Mettl3* cKO littermates (n = 24/group). (C) Spleen weight of WT and *Mettl3* cKO littermates (n = 7/group). (D) Gating strategy of B cell activation assays upon different stimulation. (E) Gating strategy of B cell proliferation assay. Data in (A-C) were presented as means ± SEM with the indicated significance (* P < 0.05; student’s t-test).

**Supplementary Figure.2**


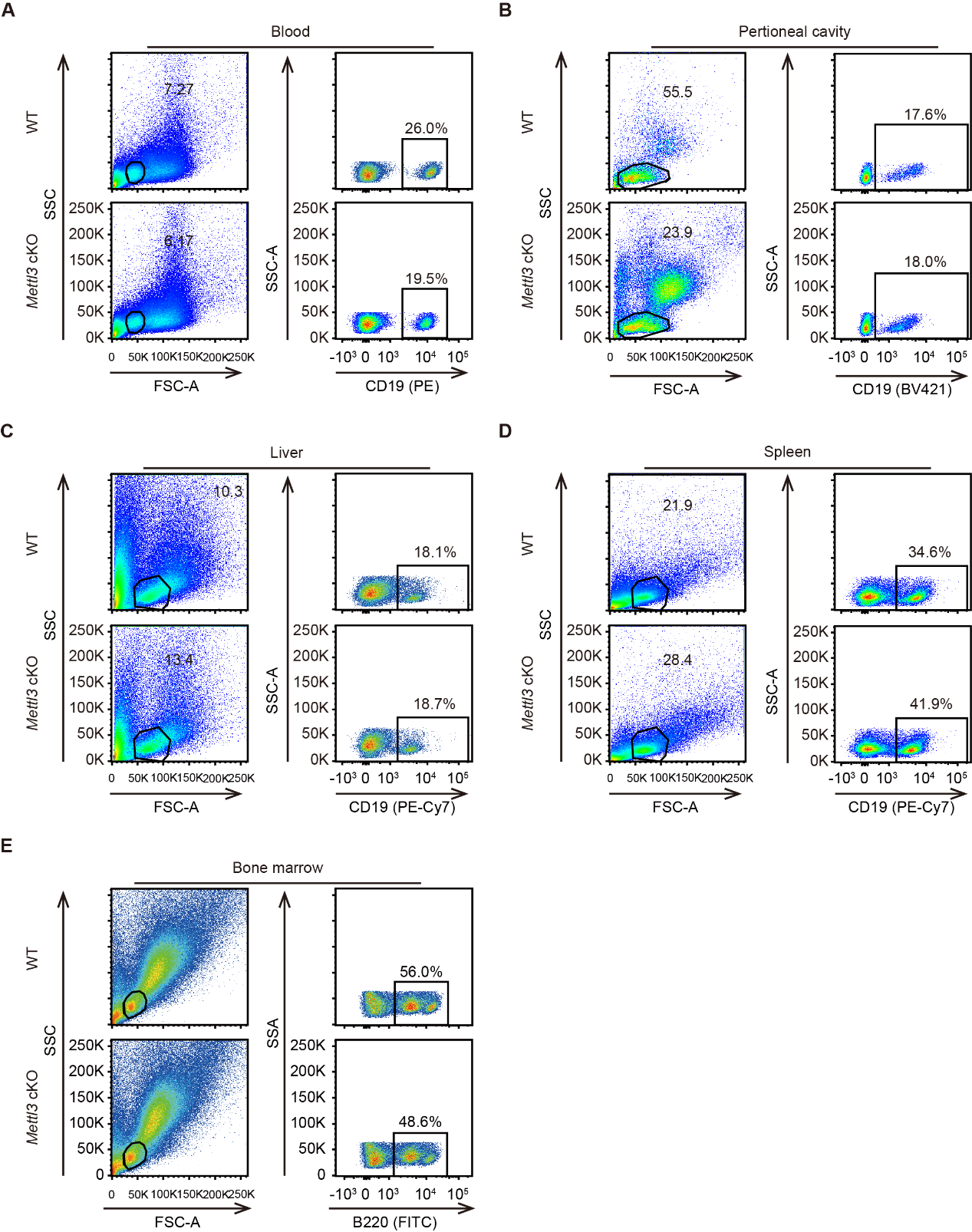


**Supplementary Figure.2. Gating strategy for B cells from different tissues. Related to Figure. 2 and Figure. 3.**

(A-E) Representative flow cytometry plots gating strategy for B cells in lymphocytes isolated from peripheral blood (A), peritoneal cavity (B), liver (C), spleen (D), and bone marrow (E).

**Supplementary table**

**1. Primers for genotyping were listed in supplementary table 1**

| **Name** | **Sequence** |
| --- | --- |
| *Mettl3*-F1 | AGAGGAGGAGAAGGTGGCAGAG |
| *Mettl3*-R1 | CCTTTCATTCACATGGCAGCAC |
| *Mettl3*-R2 | AGGCCTATAATCCTAGCACTG |
| *Cd19-Cre*-F | TATGCAGCTCCTCAGCTCCACT |
| *Cd19-Cre*-R | CTGACTTCATCAGAGGTGGCATC |

**2. Primers for RT-qPCR were listed in supplementary table 2**

| **Name** | **Sequence** |
| --- | --- |
| mouse *Mettl3_qF* | ATGAAACCTATGCCCCTCCC |
| mouse *Mettl3_qR* | TCAGGCTTTTGCTTCACCAGG |
| mouse *Tnfsf13b_qF* | ACACTGCCCAACAATTCCTG |
| mouse *Tnfsf13b _qR* | TCGTCTCCGTTGCGTGAAATC |
| mouse *Ltβ _qF* | TGGCAGGAGCTACTTCCCT |
| mouse *Ltβ_qR* | TCCAGTCTTTTCTGAGCCTGT |
| mouse *Il-10 _qF* | GCTCTTACTGACTGGCATGAG |
| mouse *Il-10_qR* | CGCAGCTCTAGGAGCATGTG |
| mouse *Tgfβ1 _qF* | CTCCCGTGGCTTCTAGTGC |
| mouse *Tgfβ1_qR* | GCCTTAGTTTGGACAGGATCTG |
| mouse *Cxcl12_qR* | GAGCCAACGTCAAGCATCTG |
| mouse *Cxcl12_qR* | CAATGCACACTTGTCTGTTG |
| mouse *Cxcl13_qR* | ATTCTGGAAGCCCATTACACA |
| mouse *Cxcl13_qR* | TTTGGCACGAGGATTCACAC |
| mouse *Ccl21_qR* | GTCCGAGGCTATAGGAAGCA |
| mouse *Ccl21_qR* | GCCCTTTCCTTTCTTTCCAG |
| mouse *Pdgfrb_qF* | TTCCAGGAGTGATACCAGCTT |
| mouse *Pdgfrb_qR* | AGGGGGCGTGATGACTAGG |
| mouse *Acta2_qF* | CTGACAGAGGCACCACTGAA |
| mouse *Acta2_qR* | CATCTCCAGAGTCCAGCACA |
| mouse *Col1a1_qF* | TGACTGGAAGAGCGGAGAGT |
| mouse *Col1a1_qR* | GTTCGGGCTGATGTACCAGT |
| mouse *Gapdh_qF* | AATGGATTTGGACGCATTGGT |
| mouse *Gapdh_qR* | TTTGCACTGGTACGTGTTGAT |

**3. Antibodies used for western blot were listed in supplementary table 3**

| **Antibodies** | **Source** | **Country** | **Identifier** |
| --- | --- | --- | --- |
| Anti B220-FITC | Biolegend | USA | 103205 |
| Anti Cd43-PE-Cy7 | Biolegend | USA | 143210 |
| Anti Cd24-PE | Biolegend | USA | 101807 |
| Anti BP-1-Alexa Fluor 647 | Biolegend | USA | 108312 |
| Anti Cd19-PE-Cy7 | BD | USA | 552854 |
| Anti Cd19-BV421 | Biolegend | USA | 115520 |
| Anti Cd5-PE | Biolegend | USA | 100608 |
| Anti Cd69-FITC | Biolegend | USA | 104506 |
| Anti Cd86-APC | Biolegend | USA | 105011 |
| Anti Cd95-PE | Biolegend | USA | 152608 |
| Anti IgD-APC | Biolegend | USA | 405714 |
| Anti IgM-PE | Biolegend | USA | 406507 |
| Anti-Desmin antibody | Abcam | England | ab15200 |
| Anti-aSMA antibody | Abcam | England | ab5694 |
| Anti-PDGFRβ antibody | Abcam | England | ab32570 |
| Anti-Collagen I antibody | Abcam | England | ab34710 |
| Anti-GAPDH antibody | Cell Signaling Technology | USA | 2118 |
| Anti-mouse IgG, HRP-linked antibody | Cell Signaling Technology | USA | 7076 |
| Anti-rabbit IgG, HRP-linked antibody | Cell Signaling Technology | USA | 7074 |

**Reagents**

| **Reagents** | **Source** | **Country** | **Identifier** |
| --- | --- | --- | --- |
| LPS | Sigma-Aldrich | USA | l2880 |
| Mouse CD40 Ligand (TNFSF5) Recombinant Protein | eBioscience | USA | 34-8512-80 |
| lgM | Jackson ImmunoResearch | USA | 115-005-006 |
| Mouse IgG Total Uncoated ELISA kit | Invitrogen | USA | 88-50400-22 |
| CFSE | Invitrogen | USA | C34554 |
| Lymphocyte separation medium | Dakewei | China | 7211011 |
| CD19 Positive Selection Kit II | Stemcell | Canada | 18954 |
| Nylon mesh (Cell Strainer) | BD Falcon | USA | 352340 |
| 0.22 μm filter | Pall | USA | PN4612 |
| 0.45 μm filter | Pall | USA | PN4614 |
| 70 μm cell strainer | BD Falcon | USA | 352350 |
| SYBR Green | Roche | Switzerland | 4707516001 |
| Fast All-in-One RT Kit | ES Science | China | RT001 |
| Collagenase IV | Sigma-Aldrich | USA | C5138 |
| Phosphatase inhibitor | Roche | Switzerland | 4906831001 |
| Complete Protease Inhibitor | Roche | Switzerland | 4693132001 |
| Immonilon ECL Ultra Western HRP Substrate | Millipore | USA | WBULS0500 |
| Carbon tetrachloride | Sigma | USA | 289116-100ml |
| Olive Oil | Macklin | China | O815211 |
| Penicillin/streptomycin | KeyGEN Biotech | China | KGY0023 |
| Triton^TM^ X-100 | Sigma-Aldrich | USA | T8787 |
| DMEM-high glucose medium | Thermo Scientific | USA | C11995500BT |
| Fetal bovine serum | ExCell | China | FSP500 |
| Tween-20 | Sigma-Aldrich | USA | P1379 |
| TRIzol | Invitrogen | USA | 15596026 |
